# Presidential inaugural address 2026: expanding computer-based testing, strengthening clinical competency assessment, and adopting artificial intelligence with caution in health professions licensing examinations in Korea

**DOI:** 10.3352/jeehp.2026.23.30

**Published:** 2026-09-10

**Authors:** Jong Han Leem

**Affiliations:** Korea Health Personnel Licensing Examination Institute, Seoul, Korea; Hallym University, Korea

On June 15, 2026, I assumed office as the 10th president of the Korea Health Personnel Licensing Examination Institute (KHPLEI) (Fig. 1). Since its establishment in 1992, KHPLEI has administered national licensing examinations for health professionals and supported license and certification management, helping safeguard the health and safety of the Korean people for 34 years.

My academic training is in preventive medicine and occupational and environmental medicine, and I previously served as dean of the College of Medicine and dean of the Graduate School of Public Health at Inha University. Throughout my career, my research and teaching have addressed issues that extend well beyond the individual clinical encounter: disparities in community health, the health consequences of the climate crisis, and environmental diseases. That experience has shaped my view of what a national licensing examination should measure. A licensing examination must validly assess not only proficiency in discrete clinical skills but also the full range of minimum competencies required of health professionals responsible for population health. Therefore, every decision KHPLEI makes should rest on evidence, and that evidence should be open to public verification. Building on this understanding and on the vision and achievements of previous presidents, I propose the following priorities for the next 3 years.

## First, we will further expand computer-based testing.

In 2025, computer-based testing (CBT) was introduced for the licensing examinations for nurse assistants, optometrists, and speech-language pathologists (level 1), bringing the number of national examinations converted from paper-based testing to CBT to 15 [1]. In 2026, the scope was extended further to include the pharmacist licensing examination, among others [2]. The nurse assistant examination is a particularly instructive case: alongside the conversion to CBT, it adopted a windowed administration model, replacing a single-day, large-scale simultaneous sitting with smaller administrations distributed across a defined period. This change has substantially broadened examinees’ choice of test dates and made the examination more convenient to take. In 2027, CBT will be introduced for the written licensing examinations for medical technologists, occupational therapists, and prosthetists and orthotists. During my term, we will advance this transition toward the longer-term goal of delivering the written components of all health professions licensing examinations through CBT by 2032.

## Second, we will expand clinical skills examinations and strengthen the assessment of clinical competence.

Accurate knowledge is essential, but so is the ability to apply it in patient care. A nationwide survey of all 40 Korean medical schools described clinical education and resources related to the Clinical Skills Test and examined faculty perceptions of its educational impact. All schools reported having a clinical skills center. Faculty respondents perceived the 2021 patient-centered revision as having less influence on clinical clerkships than the test’s initial introduction in 2009. They identified assessment of patient records and greater objectivity in standardized patient ratings as priorities for improvement [3]. KHPLEI will incorporate these proposals into its agenda for improving the Clinical Skills Test and will extend clinical skills examinations to additional professions after reviewing the readiness of the testing environment in each field.

## Third, we will periodically update job analyses, competency frameworks, and examination objectives.

The validity of an examination ultimately begins with job analysis. A recent study updating the job competencies of Korean occupational therapists derived 4 domains, 16 tasks, and 62 knowledge items through a 3-round Delphi survey of 20 experts and a focus group interview with 10 specialists, reflecting the expansion of occupational therapists’ roles from medical institutions into community settings [4]. I will establish an institutional system for periodically updating the job descriptions and examination objectives for every profession, so that examination content does not drift away from the realities of a changing health care environment. These updates should consider how changing population health needs, including health inequities and climate-related health risks, affect the entry-level competencies required of each profession.

## Fourth, we will adopt artificial intelligence cautiously and on the basis of evidence.

To prepare an assessment system suited to the era of artificial intelligence (AI), KHPLEI held an academic seminar on May 12, 2026, titled “Artificial intelligence and test development: present and future.” The seminar comprised 2 sessions, “International trends in national licensing examinations” and “The future of health professions licensing examinations.” It addressed score comparisons based on item response theory, next-generation nursing licensure examinations and technology-enhanced item types, the prediction of item difficulty and learning analytics using large language models (LLMs), and the advancement of assessment systems through AI. Building on insights from these discussions, I intend to develop the seminar into a regular forum with participation by experts from Korea and abroad.

Adoption of AI, however, demands scrutiny commensurate with the expectations placed on it. A recent study published in the *Journal of Educational Evaluation for Health Professions* (JEEHP) compared objective structured clinical examination (OSCE) stations generated by GPT-4o with teacher-written stations. Both sets were evaluated by 7 assessors who were blinded to the source of each station. All 5 teacher-written stations met the threshold for student use, whereas only 1 of the 5 GPT-4o-generated stations did. The model showed weaknesses in constructing assessment grids and generating clinically accurate images. Although the median production time was shorter with GPT-4o, the difference was not statistically significant, and any initial time saving may be offset by the expert review and revision required before use [5]. These pilot findings support a cautious approach to LLM-assisted assessment development, without establishing the performance of other models or their suitability for other assessment formats.

Accordingly, KHPLEI will consider piloting LLMs for drafting items, identifying potential content duplication, and improving linguistic clarity and consistency, with mandatory expert review of all outputs. The use of LLMs to predict item difficulty will require validation against empirical examinee response data before operational adoption. All applications will be subject to safeguards for examination security and data confidentiality.

## Continued support for JEEHP

JEEHP, the official journal of KHPLEI, has established a strong international reputation. Its 2024 CiteScore of 16.4 placed it in the 99th percentile among 1,620 education journals worldwide, and its SCImago Journal Rank of 2.068 ranked it fourth among 348 Korean journals [2]. These achievements reflect the dedication of our authors, reviewers, and editorial board members.

I ask for continued interest and participation from researchers, educators, and editorial board members. For its part, KHPLEI will actively support the sustainable development of JEEHP as a platform that advances scholarship and serves the public interest, and it will do its utmost to advance health professions education and evaluation worldwide. Throughout my term, I will work to ensure that our examinations remain valid, fair, and responsive to changing health care needs, thereby strengthening public trust and protecting the health of the Korean people.

## Figures and Tables

**Fig. 1.**
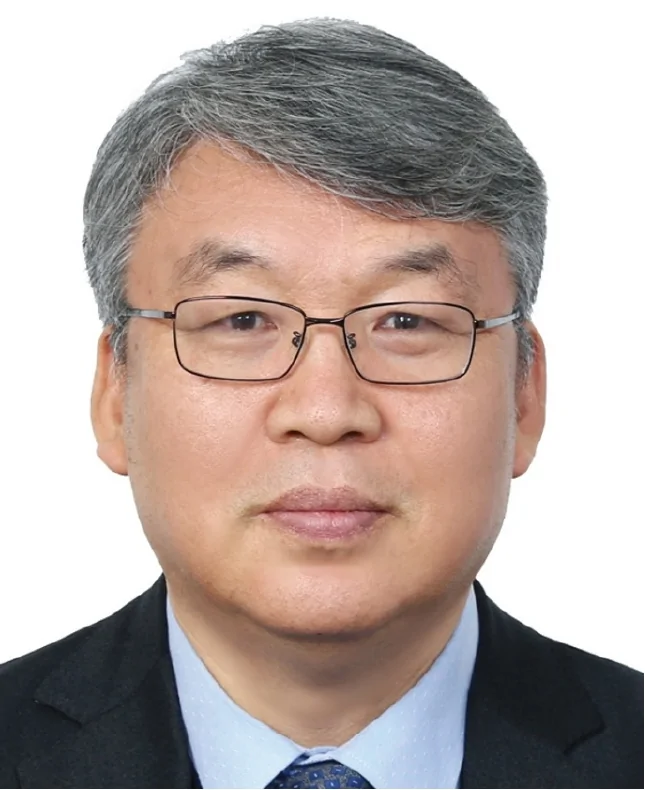
Jong Han Leem, the 10th president of the *Korea Health Personnel Licensing Examination Institute*, Seoul, Korea
